# Cellular and molecular aspects of oocyte maturation and fertilization: a perspective from the actin cytoskeleton

**DOI:** 10.1186/s40851-020-00157-5

**Published:** 2020-04-15

**Authors:** Luigia Santella, Nunzia Limatola, Jong Tai Chun

**Affiliations:** 1 Department of Research Infrastructures for Marine Biological Resources, Stazione Zoologica Anton Dohrn, Villa Comunale, Napoli 80121, Italy; 2 Department of Biology and Evolution of Marine Organisms, Stazione Zoologica Anton Dohrn, Villa Comunale, Napoli 80121, Italy

**Keywords:** Actin, Calcium, Oocyte maturation, Polyspermy, Sea urchin, Starfish, Ageing, Fertilization, Vitelline layer, Zona pellucida

## Abstract

**Abstract:**

Much of the scientific knowledge on oocyte maturation, fertilization, and embryonic development has come from the experiments using gametes of marine organisms that reproduce by external fertilization. In particular, echinoderm eggs have enabled the study of structural and biochemical changes related to meiotic maturation and fertilization owing to the abundant availability of large and transparent oocytes and eggs. Thus, in vitro studies of oocyte maturation and sperm-induced egg activation in starfish are carried out under experimental conditions that resemble those occurring in nature. During the maturation process, immature oocytes of starfish are released from the prophase of the first meiotic division, and acquire the competence to be fertilized through a highly programmed sequence of morphological and physiological changes at the oocyte surface. In addition, the changes in the cortical and nuclear regions are essential for normal and monospermic fertilization. This review summarizes the current state of research on the cortical actin cytoskeleton in mediating structural and physiological changes during oocyte maturation and sperm and egg activation in starfish and sea urchin. The common denominator in these studies with echinoderms is that exquisite rearrangements of the egg cortical actin filaments play pivotal roles in gamete interactions, Ca^2+^ signaling, exocytosis of cortical granules, and control of monospermic fertilization. In this review, we also compare findings from studies using invertebrate eggs with what is known about the contributions made by the actin cytoskeleton in mammalian eggs. Since the cortical actin cytoskeleton affects microvillar morphology, movement, and positioning of organelles and vesicles, and the topography of the egg surface, these changes have impacts on the fertilization process, as has been suggested by recent morphological studies on starfish oocytes and eggs using scanning electron microscopy. Drawing the parallelism between vitelline layer of echinoderm eggs and the zona pellucida of mammalian eggs, we also discuss the importance of the egg surface in mediating monospermic fertilization.

**Graphical abstract:**

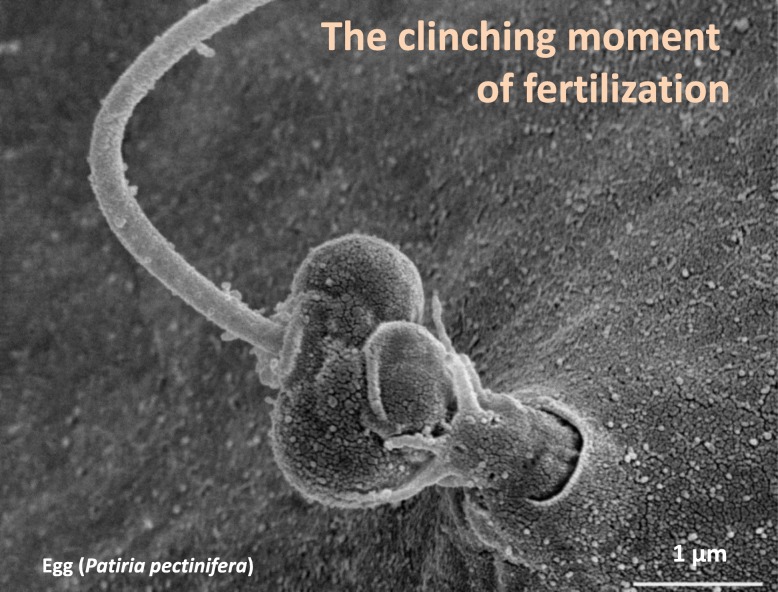

## Introduction

During gametogenesis, primordial germ-cells, which produce either eggs or spermatozoa in sexually-reproducing animals, undergo mitosis to increase in number and generate oogonia (in females) and spermatogonia (in males). With the onset of meiosis, these diploid cells are transformed into primary oocytes and spermatocytes. The meiotic (maturation) divisions that follow are markedly different in the two gametes. In male, each primary spermatocyte divides to produce four haploid spermatids, which differentiate into spermatozoa capable of fertilizing the eggs. In female, unequal partition of the cytoplasm results from two meiotic divisions that are asymmetric in size and lead to the formation of one large haploid oocyte and three polar bodies, the latter of which eventually degenerate. Another striking distinction between oocytes and spermatozoa is that the latter fertilize the eggs at the end of the meiotic divisions, whereas oocytes reach the period of fertilizability at different stages of the maturation process, depending on animal species. Indeed, marine invertebrate oocytes can be grouped into four different classes based on the meiotic stage at which fertilization takes place. Oocytes of various annelids and molluscs are fertilized at the GV stage, and the breakdown of the nuclear envelope is a sign that the oocytes have been activated. Eggs of nemerteans, ascidians, some annelids, and molluscs are blocked at the metaphase of the first meiotic division until fertilization triggers the completion of meiosis. The eggs of frogs and mammals are arrested and fertilized at metaphase II, i.e., after the second maturation spindle has formed and one polar body has been extruded [1–4]. Thus, as highlighted by Ernest Everett Just, “*the fertilizability of all animal eggs hangs together with some condition in the cytoplasm of the egg and is independent of its nuclear state, as germinal vesicle, as first or second maturation-nucleus or as a completely matured nucleus*” ([5], page 185), and may be related to the structural organization of the cortex of the female gametes.

Starfish and sea urchin eggs, which respectively are naturally fertilized before the extrusion of the first polar body and at the end of the two meiotic divisions, are useful animal models for the study of in vitro oocyte maturation, fertilization, and embryonic development. This is due to the fact that they offer advantages not only in size and abundance but also in transparency of their cytoplasm and extracellular coats. These unique properties of the echinoderm eggs allow us to perform time-lapse imaging to follow the early structural and ionic events regulating the maturation process, during which the oocytes acquire the full cytoplasmic competence to be fertilized, as well as egg activation.

**Fig. 1:**
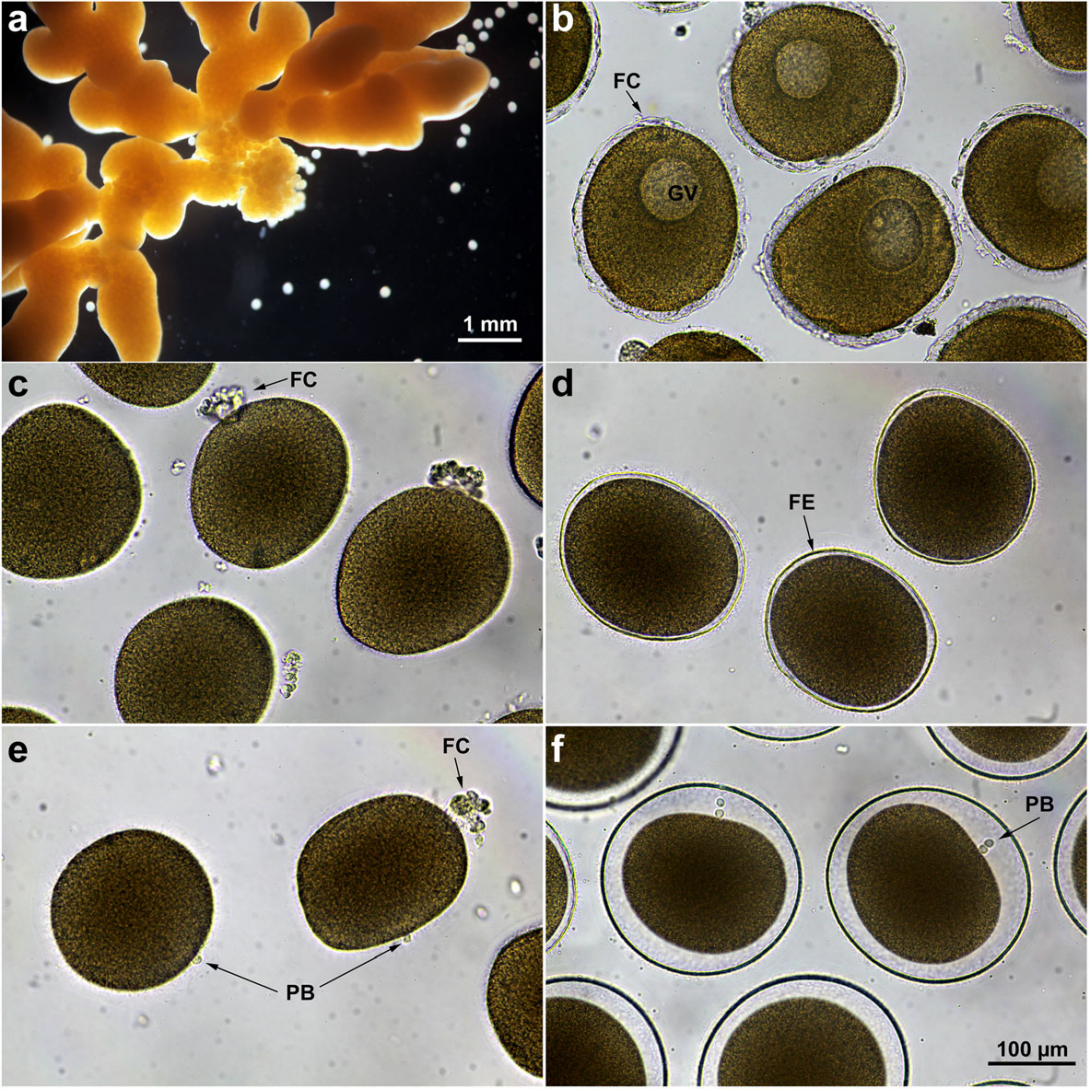
Light microscopy images of the ovary, and of living oocytes from the starfish *Patiria pectinifera* before and after 1-methyladenine (1-MA) treatment. **a** A ripe ovary dissected from *P. pectinifera,* containing numerous fully grown immature oocytes. **b** Immature oocytes isolated from the ovary are surrounded by a layer of follicle cells (FC); the large nucleus termed germinal vesicle (GV) is visible in the cytoplasm. **c** Maturing oocytes treated with 1-MA for 50 min; at this point in maturation, the FC are clustered to one side of the oocytes. This is the optimal time at which eggs can be successfully fertilized (i.e., monospermic fertilization). **d** Fertilized eggs 3 min after insemination are surrounded by the fertilization envelope (FE) as a result of the cortical granules exocytosis. **e** In the absence of fertilization, the first polar body (PB) forms 65 to 75 min after 1-MA application. **f** Extrusion of the second polar body 105 to 115 min after fertilization of eggs matured for 50 min with 1-MA (arrow)

In starfish, by the time the growing oocytes arrive at the end of the prophase of the first meiotic division, they remain blocked at that stage until spawning (Fig. 1). The release from meiotic arrest, i.e. the maturation process, is triggered by the hormone 1-methyladenine (1-MA), which is secreted by the follicles cells surrounding the oocytes, while the fully grown immature oocytes are still in the ovary [6, 7]. The hormone acts on a yet-unidentified receptor on the cell surface, and thereby induces the breakdown of the envelope of the large nucleus known as germinal vesicle (GV). The germinal vesicle breakdown (GVBD) allows the intermixing of the nucleoplasm with the cytoplasm and is followed by formation of the polar bodies (Fig. 1e arrows). The spawned maturing oocytes at sea are at the stage of their GVBD (Fig. 1c), and are to be fertilized before the extrusion of the first polar body [8, 9]. At variance with oocytes of other vertebrate species in which a second meiotic arrest may occur at different cell cycle stages, starfish eggs matured in vitro can complete meiotic divisions without further maturation arrest.

Although sperm can penetrate immature oocytes of starfish before GVBD, cortical events that block the entry of supernumerary spermatozoa and ensure normal egg activation and cleavage take place within a precise time frame only after 1-MA stimulation. Indeed, it is well known that starfish eggs lose their ability to prevent polyspermic fertilization when inseminated after being treated with 1-MA for several hours (overripe eggs). These results indicate that the competence of the egg cytoplasm to be successfully fertilized is achieved at a precise maturation stage but is lost soon after that. Studies of oocyte maturation using *Patiria pectinifera* (a.k.a. *Asterina pectinifera*, Pacific Ocean) and *Astropecten aranciacus* (Mediterranean Sea) have made interesting observations about the time frame and other requirements for eggs’ optimal fertilizability and successful development [10–12].

Recent studies have provided evidence that the cortical actin cytoskeleton is a key player in the development of mature and competent eggs manifesting normal fertilization responses. It is well established that actin, which is one the most abundant and highly conserved proteins in eukaryotic cells, participates in the maintenance of cell shape, as well as in many cellular functions such as cell migration, growth, motility, organelle movement, polarization, and exocytosis/endocytosis. Together with myosin, actin can drive not only muscle contraction, but also regulation of genes in the nucleus [13]. Actin molecules undergo transition between monomeric globular (G-actin) and filamentous (F-actin) states under the control of its own concentration and by the action of numerous actin-binding proteins (ABPs) that affect their polymerization status. Following cell stimulation, extracellular signals are often transduced through Rho family GTPases, and their downstream effector ABPs control F-actin remodelling [14]. Furthermore, because of its high-affinity binding to Ca^2+^, it has been suggested that actin may act as an intracellular buffer storing and releasing Ca^2+^ [15–17]. Consistent with this, exposure of *A. aranciacus* mature eggs at their optimum period of fertilizability to actin-depolymerizing agents, such as latrunculin A (LAT-A) and mycalolide B, triggers intracellular increases of Ca^2+^ and plasma membrane depolarization following their activation [18–20]. New knowledge has been accumulated on the roles played by actin filaments in the control of dynamic events taking place during oocyte maturation, sperm and egg activation, and cleavage. The possibility of comparing the surface morphology and the structural organization of the cortical actin cytoskeleton of polyspermic immature oocytes and overripe eggs, and their behaviour upon insemination, with those of maturing oocytes inseminated in the period of optimum fertilizability has provided insights into the importance of the egg cortical F-actin structure and dynamics in the regulation of a normal maturation and monospermic fertilization.

A number of failures in assisted reproduction technology (ART) are linked to oocytes that are fertilized not in their optimum period of maturation, but after remaining in the oviduct (in vivo ageing) or culture (in vitro ageing) [21]. It is well established that the deteriorated cytoplasmic quality of oocytes often associated with maternal age negatively affects fertilization and increases the incidence of aneuploidy for most chromosomes [22, 23], raising the risk of spontaneous abortion and obstetric complications [24]. It is important to note that postovulatory ageing in mammalian eggs induces not only alterations in the cortical actin cytoskeleton, but also structural modifications in the zona pellucida (ZP) that regulates interactions between the egg and sperm [25]. In this regard, morpho-functional data showing how the structural organization of the cortex and surrounding layers of starfish eggs undergo dynamic changes during maturation and fertilization, and how such changes are deregulated in overripe eggs, may open new perspectives on the possible ways to understand and reverse oocyte ageing for clinical applications [26].

### Actin-dependent cortical changes and the morphological alterations of the surface in the maturing eggs of starfish

**Fig. 2:**
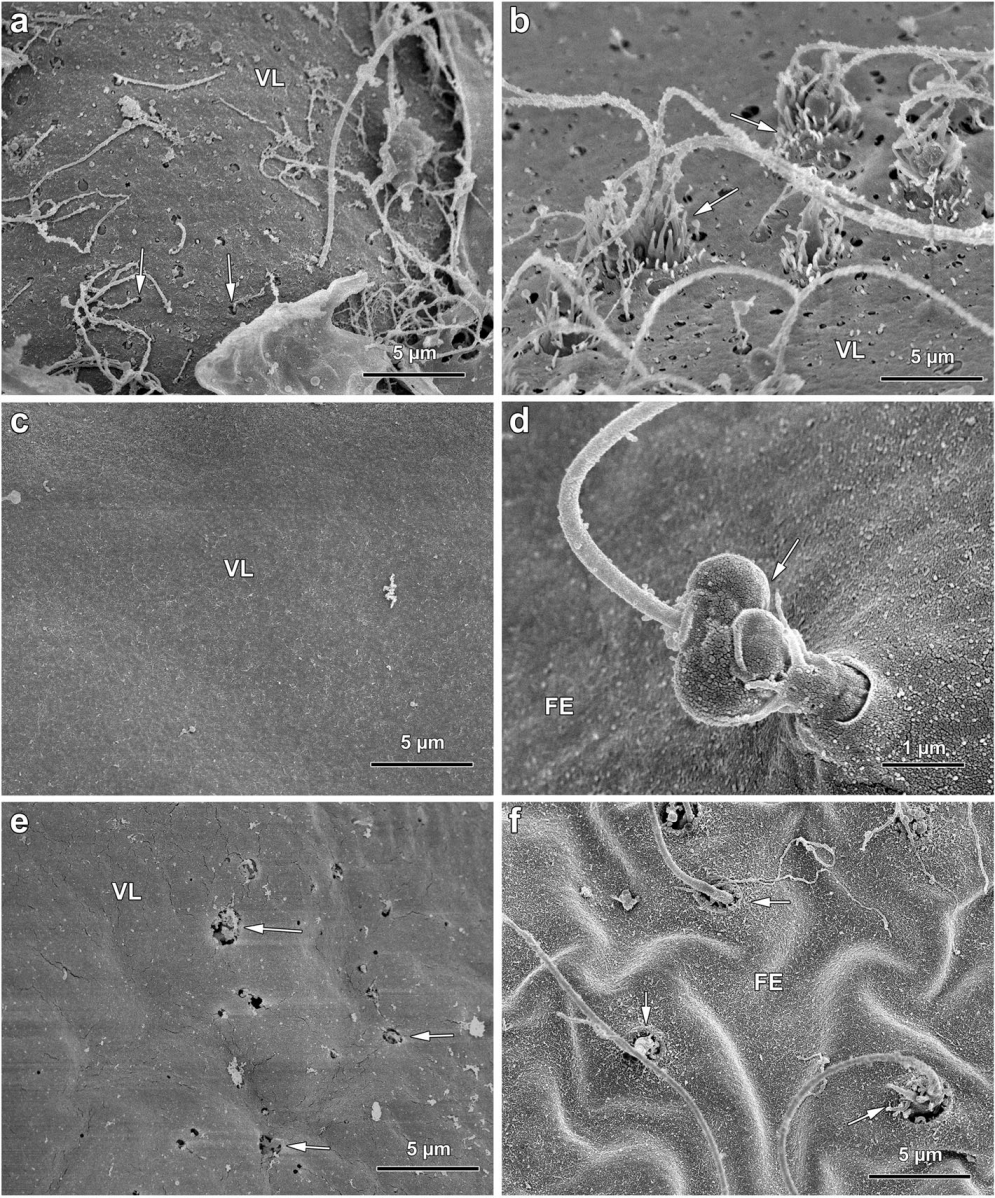
Scanning electron micrographs of the surface of immature, mature and overripe eggs of *P. pectinifera* before and after fertilization. **a** An immature oocyte showing the projections of the follicle cells (FC, arrows) penetrating the vitelline layer (VL). **b** Polyspermic fertilization of an immature oocyte. Note the formation of the fertilization cones made by a large number of long microvilli protruding from the porous VL (arrows) and capturing the multiple sperm for their incorporation. **c** An oocyte treated with 1-MA for 50 min showing the VL without the openings characteristically present in the surface of immature oocytes in A. **d** The surface of a control egg fertilized at its optimal period of maturation (50 min 1-MA treatment) shows that, at variance with the multiple sperm penetration in immature oocytes shown in B, only the fertilizing sperm is captured by cytoplasmic protrusions emanating from the fertilization envelope (FE) (arrow). Note the different structure of the fertilization cone which is formed at this stage of maturation. **e** The structural modification of the VL of the overripe eggs makes this envelope more similar to that of immature oocytes and its openings. **f** Multiple sperm penetration (arrows) through the FE in an overripe egg upon insemination

GV-stage (immature) oocytes of *P. pectinifera* dissected from the ovary (Fig. 1a) are surrounded by two layers: i) the vitelline layer, which intimately adheres to the plasma membrane, and ii) the jelly coat of approximately 20 μm in thickness, in which the projections of the outermost layer of the follicle cells (FC) are embedded (Fig. 1b). Application of 1-MA for 50 min to immature oocytes induces migration and clustering of the FC to one side of the maturing oocytes, as shown in Fig. 1c. The scanning electron microscopy (SEM) micrographs in Fig. 2 illustrate the dramatic morphological alteration of the vitelline layer (VL), which is easily visible in this species because the jelly coat is dissolved, and the cluster of FC (Fig. 1c) detaches from the maturing egg surface during the fixation procedure. Thus, in immature oocytes, cytoplasmic protrusions of the FC [27] make contacts with the oocyte plasma membrane through the pores over the VL surrounding the oocyte (Fig. 2a, arrows). About 50 min after 1-MA addition, i.e., at the optimum period for normal fertilization in this starfish species, the VL undergoes dramatic structural reorganization (Fig. 2c). These morphological changes may be essential to achieve the entry of a single sperm, since the addition of spermatozoa to eggs matured with 1-MA for 50 min always leads to monospermic fertilization (Fig. 2d, arrow) at variance with the immature oocytes, which are polyspermic at fertilization (Fig. 2b, arrows). It is interesting to note that the large number of spermatozoa penetrating the overripe eggs (treated for 6 h with 1-MA), despite the full elevation of the fertilization envelope (FE) (Fig. 2f), may represent the pathological conditions of ageing eggs that promote polyspermy. Indeed, the multiple sperm penetration in overripe unfertilized eggs may be linked to the structural modifications of the VL (the precursor of the FE) (Fig. 2e), which is more similar to that of fresh immature polyspermic oocytes (Fig. 2a and b). The modified VL structure of the overripe eggs may allow multiple sperm to interact with the egg plasma membrane, which leads to penetration of supernumerary spermatozoa through the openings on the VL (Fig. 2f, arrows). These results suggest that the changes of the VL induced by l-MA treatment at the egg surface level are sufficient to prevent or induce polyspermy by reducing or increasing interactions between sperm and the egg plasma membrane.

**Fig. 3:**
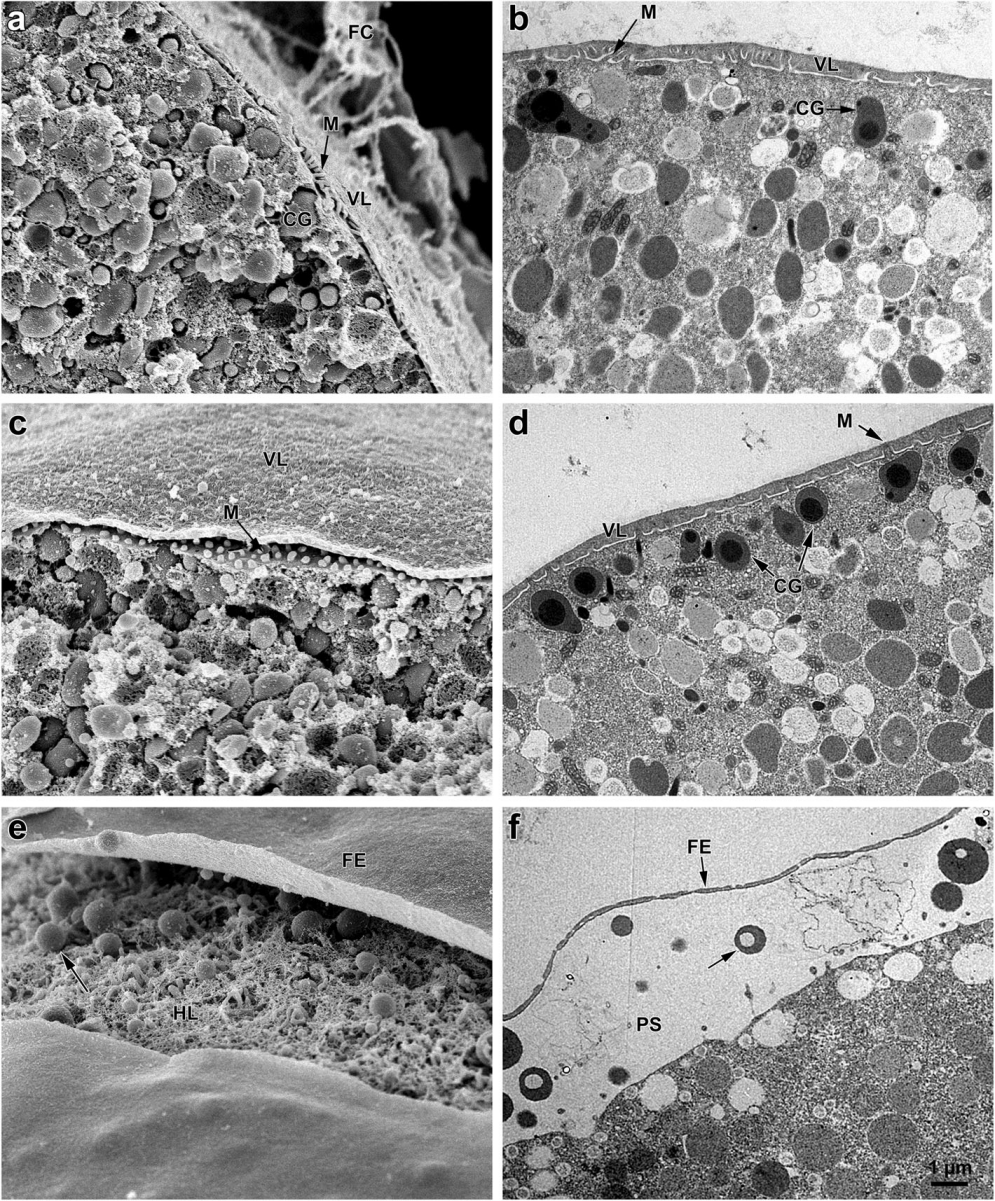
Scanning and transmission electron micrographs of immature oocytes and mature eggs before and after fertilization. **a** A fractured immature oocyte showing microvilli (M) beneath the vitelline layer (VL) and organelles in the cytoplasm. **b** Microvilli project from the oocyte’s surface into the VL. Cortical granules (CG). **c** A mature egg treated with 1-MA for 50 min. At this optimal period of fertilizability, microvilli (M) beneath the VL are shorter. **d** The ultrastructure of a mature egg stimulated with 1-MA for 50 min shows shortened microvilli (M) and cortical granules (CG) positioned beneath the egg surface. **e** Fertilization envelope (FE) elevation in a mature egg 3 min after insemination showing the spherical bodies (arrow) in the perivitelline space following CG exocytosis. Hyaline layer (HL). **f** The electron-dense spherical bodies of the CG in the egg surface before fertilization in D are now seen in the perivitelline space (PS) upon insemination. Same magnification in all figures

In addition to inducing structural modifications at the surface of oocytes, 1-MA brings about changes in their cortex that are strictly dependent on the morphological and dynamical changes of actin filaments. One to two minutes after 1-MA application, spike-like projections containing actin filaments appear on the oocytes cortex and disappear at the time of GVBD [28]. Polymerization and depolymerization of actin during starfish oocyte maturation parallel the ultrastructural changes at the surface of the oocyte, which contains organelles, cortical granules (CG), and microvilli (comprising a core of actin filament bundles). Figure 3 shows scanning electron micrographs of oocytes of *P. pectinifera* before and after maturation by 1-MA that have been subsequently fractured during fixation to visualize the oocyte surface, microvilli, CG and organelles in the cortex and inner cytoplasm. Longer microvilli are seen in the immature oocytes beneath the VL (Fig. 3a) that is penetrated by the projections from the adhering FC [29]. The porous structure of the VL ensheathing the immature oocyte is evident in the SEM micrographs of Fig. 2a. Viewed by transmission electron microscopy (TEM), microvilli are embedded in the VL covering the oocyte plasma membrane. When comparing Fig. 3a and b with c and d, it is also evident that 1-MA stimulation induces shortening of the microvilli at the time of GVBD, which represents the optimum period for a normal fertilization response. Interestingly, the structural reorganization of the oocyte cortex during maturation is accompanied by the changes in the electrophysiological properties of the oocyte plasma membrane in several animal species [30–34]. In starfish, K^+^ selective permeability diminishes following GVBD, leading to decreased conductance and less negative membrane potentials [33]. Such electrical changes have been suggested to be necessary for the generation of a more effective activation potential to electrically block the entry of additional spermatozoa [32, 35]. This hypothesis was supported by the finding that overripe and ageing *P. pectinifera* eggs, e. g., oocytes treated with 1-MA either for 120–180 or 180–240 min, display conductance much higher than the normally matured eggs and similar to that of immature polyspermic oocytes [31]. Both ageing eggs and immature oocytes are polyspermic after insemination. Our SEM images of the surface of overripe starfish eggs show an increased number of sperm spanning the FE, and show that this polyspermic penetration may be the result of the structural modification of the VL (Fig. 2e and f). Indeed, the formation of small cracks in the VL layer may allow the interactions of multiple sperm with the exposed plasma membrane regions. Thus, structural modifications of the VL together with the perturbation of the actin filaments of the underlying egg cortex may be directly responsible for the altered fertilization response and polyspermic fertilization (see below).

Other examples of oocytes showing a low resting potential state in their optimal period of fertilizability are found in mammals. They are fertilized while arrested at the second metaphase, and their meiosis is completed following the Ca^2+^ signals triggered by the sperm. However, the low resting potential of such eggs does not ensure a fast electrical block to polyspermy. At fertilization, the egg plasma membrane undergoes hyperpolarization [36], not the depolarization; this is revealed by recordings made on several mammalian species that evoke small changes in membrane potential insufficient to establish a fast electrical block to polyspermy [37]. It is well known that polyspermy-blocking mechanisms in mammals may function at the level of the extracellular ZP of the egg, which is equivalent to the VL in echinoderms. Upon fusion with the plasma membrane, the acrosome-reacted sperm induces the release of intracellular Ca^2+^ in the egg which triggers the exocytosis of the CG [38]. The release of the enzymatic contents of the CG removes the sperm binding sites from the extracellular coat by biochemically modifying the ZP glycoproteins [39]. However, in mammals, little is known about the topography or structural organization of the eggs surface at their optimum time of fertilizability and after ageing. Reduced ability to establish a membrane block to polyspermy has been observed in aged post-ovulatory ZP-free mouse eggs following the development of cytoskeletal abnormalities in the cortex [40–42]. However, in addition to the well-known age-dependent alterations of the cortical actin filaments, the possibility must be considered that the structural modifications of ZP itself in senescent eggs [43] may play an important role in eliciting an aberrant fertilization response.

During the 1-MA-induced meiosis re-initiation, besides the ionic conductance changes in the plasma membrane, the maturing oocytes of starfish undergo structural maturation—the migration and accumulation of CG towards the oocyte cortex to form a uniform layer (Fig. 3d). In starfish and sea urchin oocytes, the pre-exocytotic granules become oriented perpendicular to the egg’s plasma membrane by an F-actin-dependent process [44–46]. The separation of the VL from the egg plasma membrane at fertilization [47] follows the exocytosis of their content into the perivitelline space (Fig. 3e and f). According to the prevailing view, the formation of the FE acts as a slow mechanical block to the entry of additional spermatozoa. In line with this idea, it is accepted that immature oocytes undergo multiple sperm penetration (Fig. 2b) because of their inadequacy to undergo exocytosis to form the FE [48]. However it has been recently demonstrated that multiple sperm penetration occurs in overripe starfish eggs [11, 12], and in sea urchin eggs [49] in which the cortical actin cytoskeleton has been perturbed by actin drugs. In both cases, polyspermy can take place in the presence of a normal elevation of the FE as shown in Fig. 2f.

The mechanical and morphological changes associated with the maturation of starfish oocytes are indicated by the reduction of the stiffness of the oocytes cortex at the time of GVBD, and by increased stiffness during the formation of the two polar bodies; both of these changes in stiffness are sensitive to cytochalasin, which disrupts F-actin [50, 51]. In line with the selective cortical actin changes at the animal pole during polar body formation, a quantitative analysis has shown that actin filaments are differentially distributed in the oocyte cortex [52]. Furthermore, the two distinct pools of F-actin undergoing different polymerization/depolymerization cycles that have been characterized could account for the mechanical changes of the starfish oocyte cortex during maturation [53].

At variance with mammalian oocytes in which the GV is located in the centre of the oocyte where the meiotic spindle forms before migrating towards the oocyte cortex through a F-actin-based mechanism [54–58], the nucleus of a starfish oocyte is positioned near the surface at the animal pole by the microtubular structures and actin filaments [59, 60]. While treatment with microtubule-disrupting drugs does not modify the changes in GV morphology that precede the disassembly of the nuclear envelope, F-actin depolymerizing drugs lengthen the onset of GVBD [61]. These results highlight the important role played by microfilaments in the disassembly of the nuclear envelope and the subsequent intermixing of the nucleoplasm with the cytoplasm, a process that is essential for the changes of the egg membrane potential and for the generation of a normal Ca^2+^ response at fertilization [31, 33, 62]. Actin clustering around chromosomes has also been reported at early steps of oocyte meiosis in several species. In starfish, the very large size of the GV and the small meiotic spindle anchored to the oocyte cortex make it difficult for microtubules to capture chromosomes, and contractile actin filament networks mediate transport of chromosomes to the assembling microtubule spindle [63]. During maturation of mouse oocytes, the migration of CG and meiotic spindle to the animal pole accompanying the changes in the distribution of cortical actin is essential for the establishment of the oocyte polarity, which is indicated by the formation of a thick actin cap overlaying the meiotic spindle and by the disappearance of CG and microvilli [56, 64].

### Calcium signals during starfish oocyte maturation: role of the cortical actin dynamics

The location of 1-MA receptors in starfish oocytes has been the subject of intense investigation since microvilli are covered by a dense VL (Fig. 3b). Experiments with oocytes exposed to 1-MA after the VL was removed by trypsin [65] or separated from the plasma membrane by the divalent ionophore A-23187 [66] suggested that 1-MA acts on the oocyte plasma membrane. In line with this, microinjection of a larger amount of 1-MA into the cytoplasm of immature oocytes failed to promote maturation and GVBD [67]. The porous structure of the VL on the surface of immature oocytes (Fig. 2a) supports the idea of a direct access of 1-MA to the oocyte plasma membrane.

At variance with other animal species in which the complex signaling pathways regulating oocyte maturation have not been fully elucidated, those leading to meiosis resumption and their downstream effectors are relatively well documented in starfish. After 1-MA exposure, immature oocytes begin to form a cytoplasmic factor before GVBD occurs. The maturation promoting factor (MPF) containing cyclin-dependent kinase 1 (Cdk1) thus induces entry into meiotic M-phase by phosphorylating its substrate proteins needed for disassembly of the nuclear lamina, and thereby promotes GVBD [68]. The mediator of the 1-MA-coupled G-protein signaling pathway leading to MPF activation is phosphorylation of the protein kinase Akt/PKB, which has two main effects: i) inhibiting the Myt1 that otherwise inactivates MPF, ii) upregulating Cdc25, a phosphatase required for the activation of MPF [69]. MPF was first identified in the oocytes of the frog *Rana pipiens* by Masui and Markert [70] and later detected in a variety of organisms such as starfish, sea urchin, clams, fishes, mammals and yeast [71–73]. Cdk1 is activated by its binding to the regulatory subunit cyclin B (cyclin B/Cdk1), and inactivated through the separation from cyclin B, whose degradation occurs at the end of each M-phase. When mature oocytes of several animal species undergo metaphase arrest, MPF activity is very high and the intracellular Ca^2+^ increase at fertilization downregulates MPF and promotes completion of meiosis (see below).

The importance of Ca^2+^ signals in oocyte maturation has been established for many animal species. In starfish, 1-MA exposure could trigger a transient Ca^2+^ increase in the cytoplasm of immature oocytes and from the cell extract enriched with plasma membrane [74]. Nonetheless, the failure to detect 1-MA-induced Ca^2+^ changes in several species of starfish has led to the conclusion that GVBD and the continuation of maturation (meiotic) cycle were not Ca^2+^-dependent [75, 76]. However, results from our laboratory have shown that, in addition to a cytoplasmic Ca^2+^ transient, 1-MA induces a nuclear Ca^2+^ increase which was essential for the continuation of meiosis. Moreover, direct microinjection of the Ca^2+^ chelator BAPTA into the GV of the starfish oocyte prevented the disassembly of the nuclear envelope and the intermixing of the nucleoplasm with the cytoplasm [77, 78]. These results showed that a Ca^2+^ increase in the nucleus, not in the cytoplasm, was essential for meiosis continuation. Calmodulin (CaM) was identified as a possible downstream target of the 1-MA Ca^2+^ signals, since microinjection of antibodies against CaM directly into the GV of immature oocytes blocked meiosis progression [79]. Later studies, using more advanced imaging technology, have allowed us to detect an intracellular Ca^2+^ increase only a few minutes after 1-MA application. The increase of Ca^2+^ always starts at the vegetal hemisphere of the oocyte and is independent of its position in the chamber, as the oocyte surface was entirely exposed to the hormone. Furthermore, the Ca^2+^ transient induced by 1-MA failed to spread globally to the inner cytoplasm, but was localized to the oocyte cortex. As for the nature of the 1-MA-triggered Ca^2+^ signal, our laboratory has found that it was not linked to extracellular Ca^2+^, but strictly correlated to the structural organization of the cortical actin cytoskeleton [80]. A link between the 1-MA-induced cytoskeletal changes and the modulation of the Ca^2+^ release mediated by G-proteins has been provided by the microinjection of the non-hydrolyzable analogue of GTP and GDP. We have found that GTPγS induced a Ca^2+^ signal similar to that triggered by 1-MA, whereas GDPβS induced striking modifications of the cortical actin network and significantly inhibited the 1-MA Ca^2+^ signal [81]. The F-actin-dependent cortical Ca^2+^ increase, which runs in the oocyte cortex from the vegetal to the animal hemisphere, is followed by the subsequent increase of Ca^2+^ in the nucleus located in the latter hemisphere [80].

The universal signal for egg activation and embryo development at fertilization is an increase in intracellular Ca^2+^ following precise, species-specific, spatiotemporal patterns. The ability of an egg to produce appropriate Ca^2+^ signals at fertilization is acquired during oocyte maturation, encompassing resumption of meiosis and progression to meiosis II. In starfish, the same amount of the Ca^2+^-linked second messenger inositol 1,4,5-trisphosphate (IP_3_) microinjected into mature eggs induces a release of Ca^2+^ much higher than that elicited in immature oocytes. This higher Ca^2+^ increase does not coincide with the increased Ca^2+^ contents in the restructuring ER, but is due to the enhanced sensitivity of the Ca^2+^ stores to IP_3_ [82, 83]. The results of the studies on the global photo-activation of caged IP_3_ in the maturing oocyte have revealed that the sensitivity of the Ca^2+^ stores to IP_3_ developed with a spatial pattern similar to that of MPF, i.e., it started at the animal hemisphere and propagated towards the vegetal side [84, 85]. Studies on oocytes stimulated with 1-MA after the surgical removal of the GV have shown that the increased sensitivity to IP_3_ in maturing oocytes is correlated with the sequential activation of MPF in the cytoplasm and nucleus. Since MPF did not directly phosphorylate IP_3_ receptors (IP_3_R) as shown in other systems, an alternative hypothesis was proposed in which some components of the actin cytoskeleton may be phosphorylated by MPF and thereby produce much higher release of Ca^2+^ in response to IP_3_ [85]. Supporting the idea that the IP_3_-dependent Ca^2+^ release mechanism is modulated by microfilaments, our laboratory had previously found that microinjection of actin-binding protein cofilin into starfish eggs significantly enhances the Ca^2+^ release in response to IP_3_ or fertilizing sperm [86]. Furthermore, when mature eggs of starfish were exposed to actin-depolymerizing drug LAT-A, they exhibited intracellular Ca^2+^ increases that was reminiscent of the Ca^2+^ response in fertilized eggs [18, 20]. Interestingly, LAT-A was ineffective in inducing a Ca^2+^ response when applied to GV stage oocytes [18], which have quite different actin dynamics and cytoskeletal structures compared with the mature eggs (see below). In line with a role of F-actin in modulating the IP_3_-sensitive Ca^2+^ response, it has been shown that the sensitization of the Ca^2+^ stores to IP_3_ during oocyte maturation coincided with the ability of LAT-A to release Ca^2+^ from maturing eggs [85]. Further studies on the Ca^2+^ release mechanisms activated by LAT-A have now shown that its effect on actin filaments occurs via actin-dependent production of IP_3_ following stimulation of PLC, and it is not linked to sensitization of the Ca^2+^ stores to IP_3_ [20]. It is interesting to note that, at variance with starfish, *Paracentrotus lividus* (sea urchin) eggs exposed to LAT-A induced an increased production of IP_3_, but it did not lead to an intracellular Ca^2+^ increase [20]. These results indicate that the intracellular Ca^2+^ stores of the eggs of this species are less sensitive to the action of IP_3_, arguing against the previously suggested idea that IP_3_ is the Ca^2+^-linked second messenger produced in sea urchin eggs upon fertilization [87]. Recently, in addition to the 1-MA-triggered cortical Ca^2+^ wave that occurs a few minutes after its application, we have detected a train of Ca^2+^ spikes at the time of GVBD in the maturing oocytes of *P. pectinifera*, which are strictly linked to Ca^2+^ influx because they are inhibited when the oocytes were exposed to 1-MA in Ca^2+^-free seawater [88]. The occurrence of these cortical Ca^2+^ increases requires the presence of GV and the rearrangement of the cortical actin cytoskeleton. This actin-dependent structural modification of the oocyte cortex, concomitant with Ca^2+^ influxes at the time of GVBD, may be related to the changes in microvilli length (shortening) (Fig. 3) and the shift of the resting membrane potential in the maturing oocytes [16, 32]. The physiological significance of these Ca^2+^ influxes at the late phase of maturation was demonstrated by the finding that *P. pectinifera* oocytes matured in Ca^2+^-free seawater exhibited significantly altered actin cytoskeleton and CG distribution, as well as compromised Ca^2+^ responses and low rate of successful development following the fertilization in normal seawater [88].

Proper reorganization of endoplasmic reticulum (ER) that occurs during oocyte maturation correlates with the higher release of Ca^2+^ by IP_3_ also in the eggs of mammals. In mouse oocytes, the spatio-temporal dynamics of the cortical ER clustering at metaphase II, which is essential for the generation of normal Ca^2+^ oscillations, has been shown to be mainly mediated by microfilaments [89]. A recent comparison of the subcellular compartments of human oocytes matured in vivo (second metaphase) with those of denuded immature oocytes cultured for 6–8 h to the completion of meiosis has revealed a significant difference in the thickness of the cortical actin networks [90]. The alteration of the actin cytoskeleton in the oocytes matured in vitro may in part explain the compromised sensitivity to IP_3_ and the Ca^2+^ response at fertilization as well as low developmental competence [91].

### Contribution of actin dynamics to sperm and egg activation

**Fig. 4:**
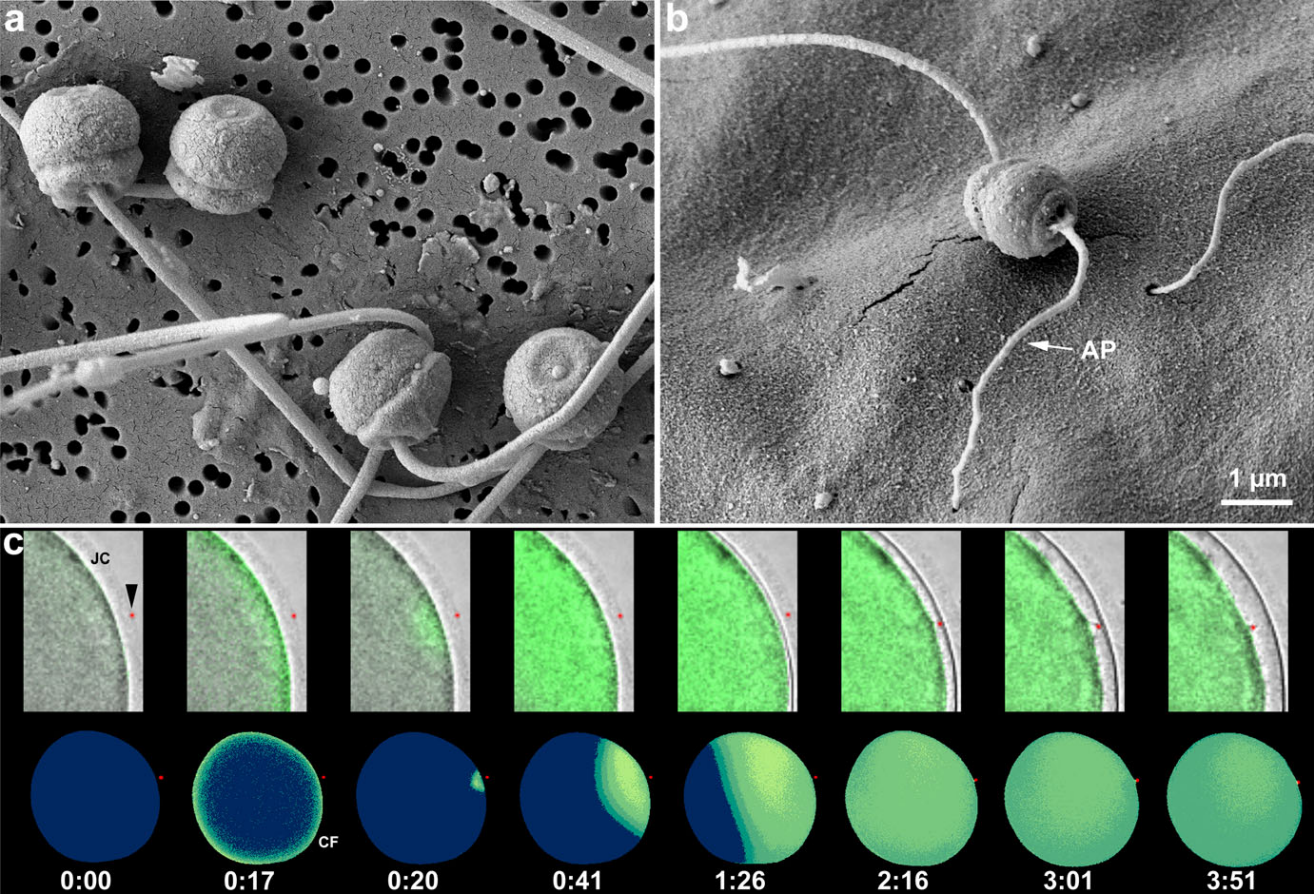
Acrosome reaction in *P. pectinifera* spermatozoa and the sperm-induced Ca^2+^ responses in the egg. **a** Scanning electron microscopy micrographs of spermatozoa fixed in natural seawater without eggs. No sign of acrosome reaction is present. **b***P. pectinifera* spermatozoa on the egg surface after acrosome reaction has taken place (see the long filamentous process, AP). **c** Ca^2+^ signals in a mature egg of *Astropecten aranciacus* following the interaction of the tip of the AP with the egg surface. A simultaneous increase of Ca^2+^ over the entire periphery of the egg (cortical flash, CF) is observed when the fertilizing sperm head (marked in red dot) is still in the outer jelly coat (JC) (t = 0:17). This Ca^2+^ increase is followed by a Ca^2+^ wave that starts from the sperm-egg interaction and propagates to the opposite side of the egg, while the head of the sperm is still far away from the egg surface (t = 0:20). Only after the Ca^2+^ wave has spread globally to the opposite side do sperm become incorporated into the egg by passing through the FE

A prior activation of the sperm by the egg’s envelopes is a prerequisite for successful fertilization [92–94]. Observations of living spermatozoa with a phase contrast microscope and the visualization of fixed samples using electron microscopy has allowed J. C. Dan to discover that the acrosome reaction (AR) in sea urchin, starfish and several other marine invertebrates is the essential mechanism for the sperm activation and the subsequent egg penetration [95]. In sea urchin, the first sperm activation is the AR which occurs when a sperm receptor makes a contact with specific sulphated fucans in the egg jelly coat [96]. This contact triggers exocytosis of the acrosome vesicle located in the apical region of the sperm, which contains bindin, an adhesive protein promoting species-specific gamete binding [97]. Simultaneously, rapid polymerization of G-actin forms the ‘acrosomal process’ [98]. The role of Ca^2+^ in the regulation of the AR in sea urchin sperm was first suggested by Dan [98], and further evidence was provided by use of the Ca^2+^ ionophore A23187 or by simply increasing the external pH in the presence of Ca^2+^. Both treatments initiated the internal polymerization of actin in the acrosomal process [99–102]. The sperm of starfish has been a particularly useful model to study the egg-jelly signaling molecules for triggering AR as well as for the role played by actin filaments in sperm-egg interaction and penetration (see below). Upon contacting the outermost jelly coat layer, a long acrosomal process (Fig. 4b) containing actin filaments is formed following simultaneous Ca^2+^ entry and pH increase in the sperm. It has been known that at least three different molecules of the starfish jelly coat act in concert to induce the structural and biochemical changes in sperm, which lead to the formation of the acrosomal process [103]. The tip of the long acrosomal process reaches the egg surface and promotes the simultaneous Ca^2+^ influx into the periphery of the egg and the propagation of the Ca^2+^ wave (Fig. 4c). All this takes place while sperm head itself is still far away from the egg surface [104, 105]. On the other hand, mammalian spermatozoa undergo a series of more complicated physiological and biochemical modifications collectively called ‘capacitation,’ while they reside inside the female reproductive tract for several hours before fertilization. Signal transduction pathways leading to actin polymerization in mammalian sperm capacitation through the activation of phospholipase D have been elucidated [106]. Very recently, a role for PIP2 and its downstream effector actin-severing protein gelsolin has been implicated in regulating sperm motility during sperm capacitation. Gelsolin, which is inactivated by its binding to PIP2, translocates from the sperm tail to the head, allowing an increase in F-actin in the tail and increased sperm motility [107, 108]. Only capacitated sperm can undergo AR which occurs during sperm penetration through the egg investments of ovulated eggs. It was shown that the binding of capacitated sperm to the egg ZP induces a Ca^2+^ influx through Ca^2+^ store depletion–activated channels. This sustained Ca^2+^ increase activates actin-severing proteins leading to depolymerization of F-actin network between the plasma and outer acrosomal membranes allowing the contact and fusion of the membranes to accomplish AR [109]. However, the site at which the AR must take place is still controversial [110]. In human sperm, other regions containing actin include the acrosomal space, the equatorial and post-acrosomal regions, and the tail.

Even if the first evidence for a sperm-induced Ca^2+^ has been produced many decades ago on experiments in fish eggs [111], the concept that the intracellular calcium increase is essential for egg activation has a long history. It has been known for a long time that “*a striking effect subsequent to insemination is the dissolution of the egg cortex*” [112], which was later shown to be due to the release of bound calcium from the protoplasmic cortex [113, 114]. This is then followed by a very rapid recovery of the ectoplasm after membrane separation [112]. The cortex dissolution starts at the site of the sperm-egg interaction and propagates as a wave along the egg surface. The cortical response of the egg, which has now been described in detail, is the result of the release of the GC contents into the perivitelline space (Fig. 3e and f) formed by the separation of the VL from the egg plasma membrane to form the FE [115]. Because other sperm cannot enter the egg in any area over which the wave has passed, Just [116] has called the reaction a “wave of negativity” which makes the egg surface refractory to the interaction with additional spermatozoa. Thus the idea that this rapid change of the cortex, which is immediately completed when the fertilizing sperm touches the egg surface, and not the separation of the VL from the egg (which occurs too late), is the fast mechanism preventing polyspermy at fertilization was originally suggested one hundred years ago [116]. Activation of the starfish and sea urchin eggs by the fertilizing sperm during or at the end of the maturation process, respectively, is marked by dramatic structural changes at the egg surface and by the concomitant alterations in the plasma membrane permeability and the intracellular calcium increase [60]. In sea urchin, electrophysiological studies have recorded depolarization of egg membrane potential 1 to 3 s after insemination as a result of increased Na^+^ permeability. This depolarization step is followed about 13 s later by a slower but longer lasting depolarization, which is called ‘fertilization potential’ or ‘activation potential’ [117–119]. In starfish and sea urchin eggs, the depolarization step and the fertilization potential precisely mirror the patterns of the Ca^2+^ influx, i.e., the cortical flash (CF), and the Ca^2+^ wave in the egg at fertilization [60]. In starfish the time lag between the Ca^2+^ influx and the onset of wave is much shorter, e.g., 2 s. The different kinetics of the electrical and Ca^2+^ changes in the two species probably reflects differences in the structural organization of their egg cortices [120]. As for the significance of this earliest electrical event at fertilization, L.A. Jaffe [35] suggested that the activation potential going positive values was responsible for the establishment of a fast block to polyspermy. While such rapid polyspermy-preventing mechanism has been suggested to act in diverse organisms [121, 122], other authors have ruled out the possibility that the fast electrical block ensures monospermy in sea urchin eggs [123, 124]. In line with this, and at variance with the prevailing view that emphasises the depolarization of the egg plasma membrane as the fast mechanism to prevent polyspermy, our laboratory has recently provided evidence that sea urchin eggs remain mostly monospermic, instead of exhibiting the expected polyspermy, when fertilized at conditions that lower the fertilization potential, i.e., artificial seawater with reduced amount of Na^+^ [125–127]. As was previously suggested ([5] on page 182), our results have shown that the observed abnormal cleavage of the eggs fertilized in low Na^+^ is due to alterations in cortical actin filament dynamics, and it is not the result of the formation of multiple mitotic spindles following aberrant microtubules organization exerted by supernumerary sperm-centrosomes [35, 128, 129]. Furthermore, the FE, which is too slow to act as a mechanical block to polyspermy, is inadequate to prevent entry of additional spermatozoa even when it is fully elevated in sea urchin and starfish eggs [11, 12, 49].

Since the sperm-induced Ca^2+^ signals activates the zygote to initiate embryonic development, one of the major problems in this field for decades has been to understand the mechanisms of the signal transduction leading to the Ca^2+^ influx and the intracellular Ca^2+^ release at fertilization [130]. In sea urchin eggs, a Ca^2+^ entry prior to the exocytosis of the cortical granules has been visualized at the cell periphery in eggs loaded with Ca^2+^ sensitive dyes [49, 131], raising the question about its role in egg activation. By contrast, later studies showed that sea urchin eggs exposed to the ionophore A23187 facilitating the transport of Ca^2+^ across the plasma membrane were able to undergo early activation and metabolic changes similar to those occurring during normal fertilization [132, 133]. Furthermore, the findings that spermatozoa whose AR had been induced by seawater containing jelly coat components released from the eggs could fertilize eggs even in Ca^2+^ free seawater [134] added weight to the intracellular origin of Ca^2+^ liberation at fertilization. However, in the experiments on the activation of sea urchin eggs by A23187, the release of Ca^2+^ from intracellular stores was judged only by the occurrence of the elevation of the VL in sea urchin eggs [132]. A comparison by Ca^2+^ imaging between the patterns of Ca^2+^ release induced by activation of sea urchin eggs by sperm or by a A23187 had not been made, unlike in the fish eggs [111]. Apart from that, recent studies on the effect of a short (5 min) application of another Ca^2+^ ionophore, ionomycin, to immature starfish oocytes have shown that ionomycin induced a dramatic rearrangement of the actin cytoskeleton in the cytoplasm and egg cortex of the oocytes that led to microvilli retraction and disruption and fusion of cortical granules with vesicles. When these ionomycin-pretreated oocytes were matured and fertilized, they gave rise to altered Ca^2+^ responses, (substantial suppression of the CF and delayed Ca^2+^ wave) and inhibition of the CG exocytosis [135]. The results showed that ionomycin compromised the fertilization response and subsequent cleavage by inducing non-physiological F-actin-based changes of the egg cortex [29].

In mammals, the fertilizable oocyte is arrested at metaphase of second meiotic division due to the high activity of MPF and of the MAPK kinase, (M-phase activating protein kinase), and inhibition of anaphase promoting complex or cyclosome (APC/C) [136]. Following insemination, the sperm-induced Ca^2+^ signals triggers inactivation of the two kinases, transition from metaphase to anaphase by the CaMKII-dependent activation of APC/C, inactivation of MPF, and initiation of embryo development [137]. At fertilization of mammalian oocytes, it is widely accepted that a series of spatio-temporal Ca^2+^ oscillations is triggered upon delivery of a soluble sperm-specific factor identified as a PLCζ that enters the egg upon gamete fusion [138, 139]. That PLCζ promotes IP_3_ formation through the hydrolysis of PIP2 and Ca^2+^ release upon IP_3_-gated opening of IP_3_Rs on the ER has been claimed by experiments in which the microinjection of anti-IP_3_Rs antibodies inhibited the sperm-induced Ca^2+^ release [140]. At variance with PLCδ1, PLCζ lacks an N-terminal pleckstrin homology (PH) domain [141] and thus has only the ability to hydrolyse the PIP_2_ substrates located in intracellular membranes rather than the plasma membrane [142]. Analysis on the absence of PLCζ in the sperm of infertile patients who failed to obtain activation of the oocyte following intracytoplasmic sperm injection (ICSI) has supported the significant role for PLCζ in the egg activation process. However, recent studies on oocytes activated using either in vitro fertilization (IVF) or ICSI by sperm derived from *Plcz1*-null mice have shown the absence of the typical Ca^2+^ oscillatory pattern required for oocyte activation. Nevertheless, a small number of zygotes fertilized by the sperm genetically lacking PLCζ were able to develop to blastocyst, and to produce live offspring [143]. In another study, it has been found that *Plcz1* KO mice failed to induce Ca^2+^ changes following ICSI, but still elicited a Ca^2+^ increase following IVF. The altered pattern of Ca^2+^ release suppressed embryonic development, probably due to the failure of proper oocyte activation or to the polyspermy induced by the absence of adequate Ca^2+^-linked block mechanisms against the entry of supernumerary sperm [144]. These results suggest that the existence and a role of a PLCζ-independent Ca^2+^ release mechanism(s) at the oocyte surface are yet to be understood in mammalian oocytes, and raise some concerns in that, despite the essential role of PLCζ in inducing Ca^2+^ oscillations to ensure monospermic fertilization in mice, the ICSI protocol bypasses what normally occurs at the oocyte cortex following sperm-egg fusion. In this regard, it is noteworthy that the cortices of mouse and ascidians oocytes are highly reactive and structurally organized to facilitate the initiation of Ca^2+^ oscillations. This has been demonstrated by the visualization of the Ca^2+^ signal that originates from the periphery of these oocytes after ICSI and then propagates across the cytoplasm [145]. In line with a role of a cortical Ca^2+^ release in the regulation of a normal oscillatory Ca^2+^ response, the Ca^2+^ imaging analysis of intact *Ciona intestinalis* ascidian oocytes (with chorion and accessory cells attached) shows a CF, resulting from Ca^2+^ influx, which precedes the Ca^2+^ oscillations that is usually undetected if the chorion and neighbouring cells are removed prior to fertilization [146].

In line with a role of Ca^2+^ influx in the initiation of the sperm-induced Ca^2+^ signals and polar body emission, recent studies on ZP-free mammalian oocytes have shown that, STIM and Orai-linked store-operated Ca^2+^ entry mechanism is not required to sustain the persistent sperm-induced Ca^2+^ oscillations [147]. However, the mechanosensitive calcium and magnesium –permeable transient receptor melastatin 7 (TRPM7), and the CaV3.2 T-type channels have been identified as key regulators of Ca^2+^ influx following fertilization of mouse oocytes [148]. These results suggest that the amount and pattern of the Ca^2+^ entry via mechanosensitive activation of TRP-type cation channels during fertilization of mammalian oocytes may be heavily dependent on the structural integrity of the egg and appropriate tension controls by the cortical actin cytoskeleton [149, 150].

Interestingly, in *Drosophila*, it has recently been reported that an intracellular Ca^2+^ increase following egg activation, which is independent of fertilization but triggered by mechanical pressure in the female reproductive tract, is initiated by entry of extracellular Ca^2+^. This cortical Ca^2+^ increase starts at one pole of the egg and initiates the Ca^2+^ wave that propagates to the opposite one. The initial Ca^2+^ influx is mediated by the opening of mechanosensitive ion channels indicating a conserved mechanism of calcium signaling in egg activation between protostome (*Drosophila*) and deuterostome (mouse) [151]. Furthermore, a relationship between changes in the actin cytoskeleton and the Ca^2+^ increase at egg activation has been demonstrated by showing that reorganization of the actin cytoskeleton that covers the cortex of the mature eggs is required for the Ca^2+^ wave upon egg activation [152].

As to the clinical applications, Ca^2+^-ionophore treatment, which is currently used to restore the lack of normal sperm-induced Ca^2+^ response in human oocytes [153, 154], is among the methods being used for assisted oocyte activation in ICSI. However, there are presently no data reporting that its application induces the Ca^2+^ oscillations that are critical for successful oocyte activation and the high quality blastocyst formation [155, 156]. The Ca^2+^-ionophore, which elicits a single Ca^2+^ increase, thus not mimicking the Ca^2+^ oscillation at natural fertilization [157], may still lead to proteolysis of cyclin B and to a reduction in the CDK1 activity. However, the amount of Ca^2+^ released may not ensure that these events occur and persist. In human oocytes, about 10 to 20 Ca^2+^ transients occurring at intervals of 30–60 min are the physiological pattern associated with successful development. The oscillatory pattern (amplitude and frequencies) of Ca^2+^ increases appear to have no effects on early cleavages, implantation, and post-implantation development, but foetal morphology [158] and weight variations in offspring have been reported to be affected by abnormal Ca^2+^ signals [159]. This, together with the notion that Ca^2+^ ionophores can induce disintegration of the cytoplasm with prolonged exposure and severe structural modifications of the egg cortex in several species [132, 135, 160, 161], raise concerns about their use in clinical IVF.

### The actin cytoskeleton in the Ca^2+^ signaling pathways at fertilization in echinoderms

Sea urchin gametes have been widely used to study the role of Ca^2+^ signaling pathways in the fertilization process and development. These eggs have completed the meiotic cycle and await the sperm to activate their developmental program. Microinjection of Ca^2+^ dyes into the cytoplasm of intact unfertilized eggs allows the visualization of the sperm-induced Ca^2+^ increase, which occurs in the form of a subitaneous release of Ca^2+^ at the periphery of the egg [130, 162]. This is the result of the opening of voltage-sensitive Ca^2+^ channels following depolarization of the egg plasma membrane. A Ca^2+^ wave follows that starts at the sperm-egg interaction site and propagates to the opposite pole. The role of a Ca^2+^ influx from external seawater [163] has been neglected for decades as the eggs could be activated in seawater containing low Ca^2+^ [164], or even in Ca^2+^ free seawater by acrosome-reacted sperm [134]. Investigations aimed at understanding how fertilization activates Ca^2+^ release in echinoderm eggs have pointed to the exclusive role of IP_3_ as the messenger that initiates the increase and spreading of Ca^2+^. This possibility emerged from experiments in which the measurement of phosphoinositide turnover and IP_3_ generation were found to coincide with the sperm-elicited Ca^2+^ wave [87], and by the finding of the blockade of the Ca^2+^ increase by chemical inhibitors of PLC [165, 166]. Additionally, when PLC activation was blocked, the sperm-induced Ca^2+^ wave was found to be altered [167, 168]. However, the IP_3_R antagonist heparin was found to abolish the sperm-induced Ca^2+^ wave only when it was co-injected with antagonists of ryanodine receptors (RyRs), while a massive amount of Ca^2+^ was mobilized by the microinjection of ryanodine and cyclic ADP-ribose (cADPr). These Ca^2+^ signals regard only the Ca^2+^ wave taking place at fertilization, and not the initial cortical Ca^2+^ influx. They were nevertheless used to propose an exclusive coupling mechanism between IP_3_ and RyRs in shaping the Ca^2+^ signaling at fertilization of sea urchin eggs [169, 170]. However, the finding that cADPr and its upstream modulator cGMP were increased prior to the generation of the Ca^2+^ transient cast doubt on the notion that IP_3_ alone initiates Ca^2+^ release at fertilization [171]. Additional studies on sea urchin eggs have shown that a third Ca^2+^-linked second messenger, NAADP, mobilizes Ca^2+^ from stores localized in acidic vesicles in the cytoplasm of intact eggs, which were insensitive to IP_3_ and cADPr [172]. It was later hypothesized that the receptor of NAADP was the two-pore channel (TPC), a member of the superfamily of voltage gated channels located in endolysosomal structures [173].

In starfish, the global photo-activation of caged NAADP pre-injected into the cytoplasm of oocytes and eggs elicited a Ca^2+^ increase from Ca^2+^ stores confined to the cortex, which then spread to the cell centre. However, the NAADP-induced Ca^2+^ increase was inhibited when the uncaging was performed in Ca^2+^-free seawater [174]. Electrophysiological studies then provided evidence that NAADP targeted a Ca^2+^ permeable ion channels on the plasma membrane. NAADP was shown to trigger plasma membrane depolarization and was thus suggested to initiate sperm-induced Ca^2+^ influx that produced the cortical Ca^2+^ influx [62]. Interestingly, the NAADP-triggered Ca^2+^ influx was shown to be dependent on the integrity of the cortical actin cytoskeleton, as judged by the modulation of Ca^2+^ currents by actin drugs [175]. The possible involvement of acidic vesicles in eliciting the NAADP-induced Ca^2+^ entry in starfish oocytes, as suggested for sea urchin eggs, was assessed pharmacologically using drugs known to disrupt acidic compartments. However, the treatment failed to inhibit the NAADP-induced plasma membrane depolarization [176]. Recently, knockdown of all three TPC channel isoforms expressed in the starfish *Patiria miniata* showed that Ca^2+^ signals at fertilization are only slightly altered, raising doubts about the contribution of these channels to the initiation of the Ca^2+^ response at fertilization [177].

Starfish gametes have greatly contributed to the understanding of the signaling pathways leading to the intracellular Ca^2+^ elevation following fertilization. A normal CF (shorter than that elicited in sea urchin eggs) and the Ca^2+^ wave occur when the sperm fertilizes the maturing starfish oocytes after GVBD and before the extrusion of the first polar body [60, 177]. The IP_3_-dependent nature of the sperm-induced intracellular Ca^2+^ wave was demonstrated by experiments in which the microinjection of the SH2 domain of PLCγ delayed the initiation of the Ca^2+^ wave, leaving the CF unaffected [20, 168]. However, microinjection of heparin into a fertilizable starfish (*A. aranciacus*) egg completely abolished CF, in addition to lowering the amplitude of the multiple, but abortive, intracellular Ca^2+^ waves that resulted from polyspermic egg [104]. It was later shown that this abnormal fertilization response was the consequence of the alteration of the structural organization of the cortical actin cytoskeleton [104]. The F-actin-linked morphological changes of the egg cortex induced by heparin favoured polyspermic fertilization and inhibited CG exocytosis not only at fertilization, but also in response to the Ca^2+^ releases triggered by uncaging of IP_3_ and cADPr [80, 104]. Thus, at variance with previous reports that cytoplasmic Ca^2+^ [178] is sufficient for CG exocytosis [179], our results have shown that the integrity of the actin cytoskeleton of the starfish egg cortex is also essential for successful CG exocytosis. In line with this, it has been recently found in mouse eggs that the clearance of the cortical actomyosin layer during CG exocytosis is necessary for the access of the CG to the plasma membrane [180].

**Fig. 5:**
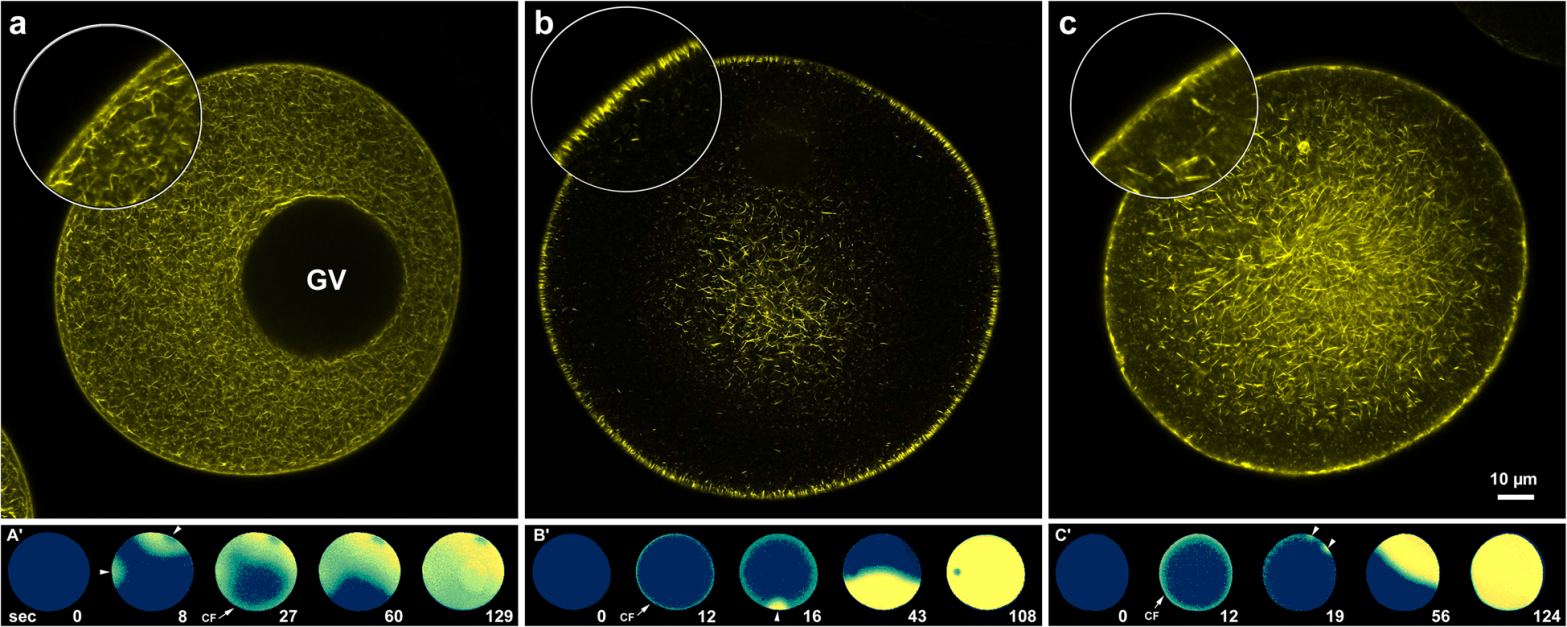
F-actin staining in living *A. aranciacus* oocytes at different maturation stages and calcium responses upon insemination. **a** Confocal image of the F-actin in an immature oocyte (GV-stage). A network of actin filaments is visible in the cytoplasm of oocytes not treated with 1-MA. **b** Reorganization of F-actin following 1-MA stimulation: The cytoplasmic F-actin network seen in **a** is no longer visible. At this stage of maturation (70 min of hormonal treatment) the actin fibers are perpendicular to the egg surface. **c** Alteration of the cortical actin in an overripe egg (1-MA treatment for 6 h) as evidenced by the irregular layer of the cortical F-actin as compared to that in **b**. Note the absence of the actin fibres perpendicularly oriented at the egg surface. **a’** Ca^2+^ response of an immature polyspermic oocyte upon insemination. Two Ca^2+^ waves (arrowheads) that converge to the centre of the oocyte precede the release of Ca^2+^ in the oocyte cortex (CF, arrow). **b’** Egg treated with 1-MA for 70 min experience a normal Ca^2+^ response with a CF (arrow) which precedes the single Ca^2+^ wave. **c**′ Two Ca^2+^ waves (arrowheads) as a result of polyspermic fertilization in overripe eggs inseminated 6 h after hormonal treatment

A recent investigation by our group on the effects of ageing on cellular and molecular events taking place in fertilized eggs of *A. aranciacus* (starfish) showed that a prolonged 1-MA-induced maturation (6 h instead of 70 min, overripe eggs) of freshly collected immature oocytes promoted pathological polyspermy at fertilization [12]. The state of the cortical and cytoplasmic F-actin in the GV stage oocytes, mature fertilizable eggs, and overripe eggs are shown in the confocal microscopic images (Fig. 5). The network of actin filaments widely distributed in the cytoplasm of immature oocytes (Fig. 5a) is no longer visible after the treatment with 1-MA for 70 min, while their presence is now prominent near the plasma membrane with the actin fibres oriented perpendicular to the egg surface (Fig. 5b). However, this orderly arrangement of the actin filaments subjacent to the plasma membrane, which is characteristic to mature eggs (Fig. 5b), is now lost in overripe eggs (Fig. 5c), indicating a dramatic reorganization of the cortical F-actin during egg ageing. The structural organization of the cortical F-actin at different maturation stages and its response to fertilizing sperm determines the pattern of the Ca^2+^ increases at fertilization. Indeed, the GV stage oocytes respond to insemination by eliciting multiple Ca^2+^ waves (arrowheads) that precede the occurrence of the CF (Fig. 5a’). The Ca^2+^ waves then converge to the centre of the oocyte. By contrast, eggs treated for 70 min with 1-MA, which represents the optimum period to achieve monospermic fertilization, respond to fertilizing sperm by forming a CF followed by a single Ca^2+^ wave (arrowhead) running from the sperm-egg interaction site to the opposite pole (Fig. 5b′). In overripe eggs, the CF is followed by multiple Ca^2+^ waves (arrowheads) as a result of polyspermic fertilization (Fig. 5c′).

Our and previous findings that overripe eggs are penetrated by numerous spermatozoa at fertilization despite the elevation of the FE [10–12] have suggested that some mechanisms other than CG exocytosis may finely control the entry of only one sperm. One explanation for multiple sperm penetration could be morphological modifications of the VL (Fig. 2e) on the surface of overripe eggs, which may allow interactions of multiple sperm with the egg plasma membrane through the numerous openings in the egg envelope. The concomitant alteration of the cortical F-actin structure in the overripe eggs may impair its rapid changes at fertilization, which would otherwise prevent the binding and penetration of additional spermatozoa.

In mammals, aged eggs also show cellular and molecular changes that compromise the fertilization response and optimal development [42]. As with overripe eggs of starfish, the structural modification initiates at the ZP [25], which becomes harder and requires longer time for enzymatic digestion [181]. The structural abnormalities of the egg cortex include altered topography of the plasma membrane which is not homogenously covered by microvilli [182] and thus may be responsible for the abnormal fertilization response [42].

The roles played by the egg cortical actin cytoskeleton in regulating a normal fertilization reaction have also been demonstrated in sea urchin. Analysis of the effects of actin drugs promoting F-actin depolymerization or stabilization on the fertilization reaction of *P. lividus* eggs has shown that modification of actin structure and dynamics led to alterations of the sperm-induced Ca^2+^ release, pattern of sperm entry, and the extent of the FE elevation [49]. The characteristic rearrangement of actin fibers in the cortex of monospermic eggs and their concerted translocation towards the center of the zygotes [183] were also inhibited when *P. lividus* eggs were fertilized in low sodium media. As a result, these monospermic zygotes underwent aberrant cleavages [125]. Seawater salinity changes also alter cortical actin dynamics and the structure of the cytoplasm and organelles , which lead to alteration of the sperm-induced Ca^2+^ signals and reduced success rate of development (Limatola N, Chun JT, Santella L. Effects of salinity and pH of seawater on the reproduction of *Paracentrotus lividus,* submitted). Similarly, treatment of sea urchin eggs with agents that disrupt, relocate, or fuse cortical vesicles and CG also alter microvillar morphology and density, and these eggs exhibit altered Ca^2+^ kinetics at fertilization in its rise and fall [184]. The crucial role played by actin-dependent microvilli morphology and the actin-associated cortical acidic vesicles in generating normal fertilization reactions has been further provided in the eggs whose cortical vesicles are dislodged from the egg plasma membrane by physical force [184–186]. Furthermore, our findings in starfish on the functional importance of the changes in actin filament organization of the unfertilized egg cortex in regulating monospermic fertilization have been extended to sea urchin as well [49]. Very recently, we found that nicotine induces polyspermic fertilization through a mechanism that is independent of the cholinergic pathways but is mediated by changes in the egg cortical F-actin meshwork and its altered polymerization dynamics [187].

Within a few minutes after fertilization, the cortical actin cytoskeleton visibly reorganized at the egg surface from the point of sperm entry toward the entire egg surface [188, 189]. The cortical actin polymerization coincides with an efflux of acid into the surrounding seawater [190] and with the elongation of microvilli into the perivitelline space [191, 192]. Actin bundle formation and full elongation of microvilli require Ca^2+^ elevation and the action of Ca^2+^-sensitive actin binding proteins [193–196]. Furthermore, the normal equidistant elevation of the FE depends on the formation of actin-containing spikes protruding from the egg surface into the perivitelline space [197]. Cortical actin reorganization following fertilization also includes detachment and translocation of actin filaments from the egg surface towards the centre of the zygote, which is a prerequisite for a normal cleavage [125, 135, 183].

### Actin-based morphology of the fertilization cone in normal and pathological eggs

**Fig. 6:**
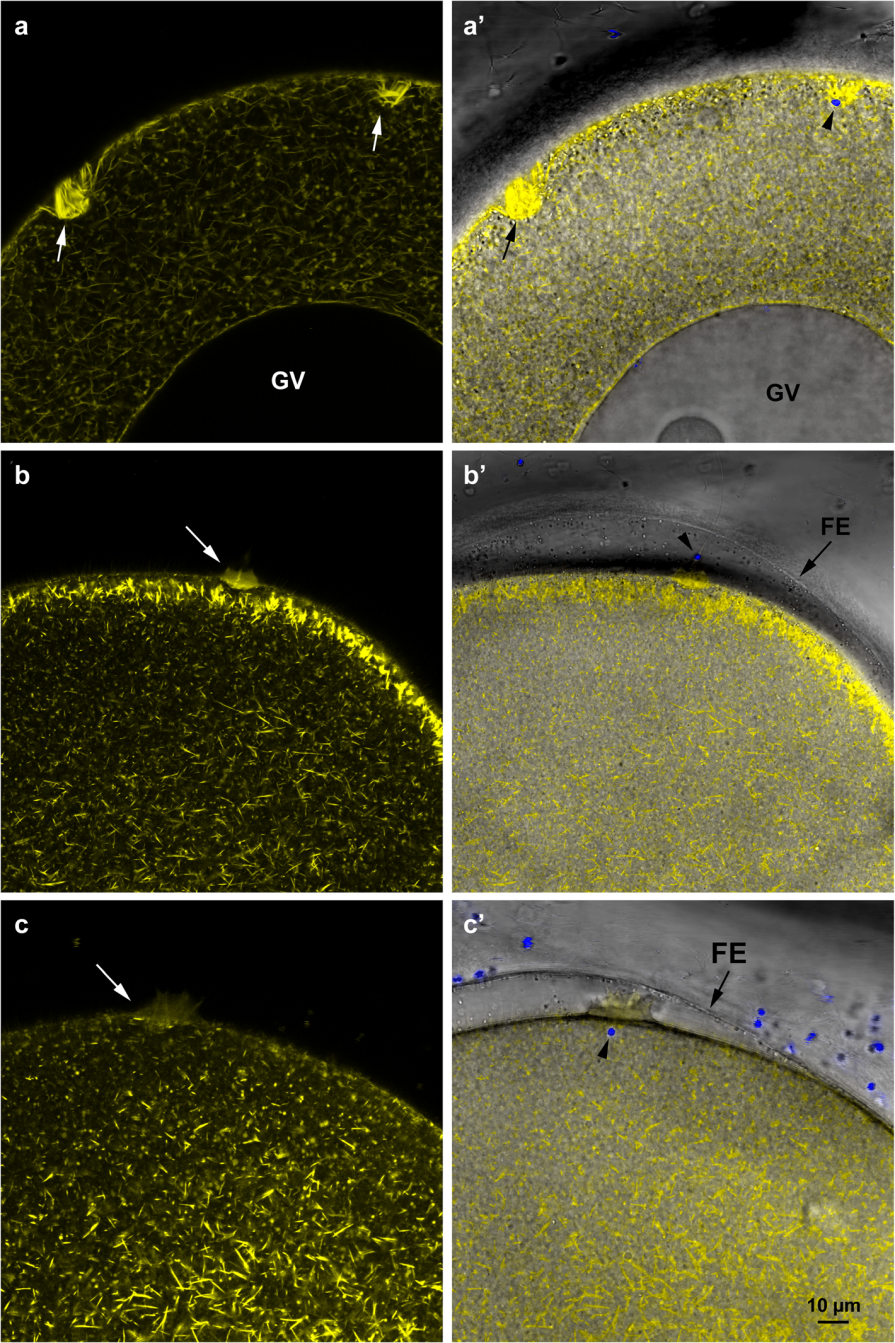
Confocal images of the cortical actin cytoskeleton in starfish oocytes fertilized at different meiotic stages. Immature oocytes, mature eggs and overripe eggs of *A. aranciacus* were microinjected with Alexa Fluor 568 phalloidin. **a** F-actin staining of the fertilization cones in a polyspermic immature oocyte. Note the large number of actin bundles composing the fertilization cones that will incorporate the sperm in the absence of a FE elevation shown in the overlay image in **a’**. **b** A mature monospermic egg fertilized in its optimal stage of maturation (70 min 1-MA treatment) that shows a fertilization cone. Note the initiation of the centripetal translocation of individual actin fibres at the subplasmalemmal zone and the elevation of the FE in the overlay image in **b′** as a result of the CG exocytosis. **c** Reorganization of the F-actin in an overripe egg fertilized after 6 h hormonal treatment. Note that the centripetal translocation seen in fertilized mature eggs is absent in overripe eggs upon insemination. **c**′) Image overlay showing the fertilization envelope (FE) elevation and incorporation of sperm in the egg (arrowhead). Multiple spermatozoa enter even in the presence of normal elevation of FE

The interaction between the fertilizing sperm and sea urchin eggs occurs at the tip of microvilli containing bundles of actin filaments which may participate in sperm–egg binding and fusion [198]. A large increase in the mass of actin filaments occurs at the site of sperm entry, forming the fertilization cone. This cytoplasmic protrusion has been observed by TEM [193] and in living eggs by fluorescent actin or phalloidin [183, 189]. In sea urchin and starfish, the fertilization cone made by actin filaments engulfs and incorporates the sperm into the eggs [199, 200]. Fertilization of GV stage starfish and sea urchin oocytes leads to formation of multiple fertilization cones (polyspermic fertilization) and fail to produce a FE, which is well visible in the light microscope [11, 193]. Their fertilization cones exhibit abnormal length presumably due to the different actin organization of the cortex, as judged by the comparison with the mature eggs that are normally monospermic at fertilization. The SEM images (Fig. 2) show that the fertilization cone in the polyspermic immature oocytes and overripe eggs consist of a larger number of elongated microvilli protruding from the physiological, and pathological, porous structure of the VL, respectively. The microvillar structure (finger-like protrusions) of the fertilization cones following polyspermic fertilization (Fig. 2b) is dramatically different from the cytoplasmic protrusion emanating through the pore of the FE during monospermic fertilization (Fig. 2d). Differences in the shape of the fertilization cones in immature oocytes, mature eggs, and overripe eggs are also evident when F-actin is stained and viewed with confocal microscopy. As shown in Fig. 6a, in polyspermic GV stage oocytes multiple fertilization cones containing actin filaments are formed. Once the spermatozoa are incorporated into the oocytes, they tend to remain localized in their cortical regions (Fig. 6a’, arrowhead). By contrast, a mature monospermic egg of starfish forms one single fertilization cone on the egg surface upon insemination (Fig. 6b and b’), the morphology of which is different from the ones produced by immature oocytes (Fig. 6a). The cytoplasmic protrusions of actin bundles previously shown in confocal and epifluorescence images [12, 197] appear to traverse the FE [104, 105] and ‘grab’ the head of the fertilizing sperm for its incorporation (Fig. 2d). The confocal image of F-actin at the cortex of a mature egg following monospermic fertilization shows the cytoplasmic translocation of well-defined actin filaments (Fig. 6b) that start to migrate to the centre of the egg. Figure 6b’ shows the elevation of the FE as well as the fertilization cone that incorporates the incoming sperm (arrowhead). The alteration of the cortical actin in eggs fertilized 6 h after hormonal treatment (overripe eggs) compromises the fertilization response (Fig. 6c). In addition to the formation of a fertilization cone that is morphologically different from that of monospermic eggs, translocation of actin filaments from the egg surface to the centre fails to occur presumably because actin fibres are disarrayed in the cortex of unfertilized eggs (Fig. 5c).

At variance with echinoderm eggs, the involvement of actin filaments for sperm incorporation in mammalian fertilization is controversial. This is probably due to the fact that different actin drugs have been used to depolymerize or stabilize the cortical actin in eggs before fertilization. These controversial results may be attributable to the specific and diverse mechanisms of action of actin drugs, which complicates comparison of their effects in perturbing the actin polymerization step [56].

## Conclusion and perspectives

Dynamic remodelling of the actin cytoskeleton is essential not only for the control of cell shape but also for the regulation of important cellular processes. An increasing body of evidence suggests that the structural organization of the actin cytoskeleton and its physical changes induced by external signals dynamically regulate a large number of events occurring during oocyte meiotic maturation and fertilization. During maturation, the acquisition of competence of the oocyte to be successfully fertilized is in major part controlled by the actin filaments of the egg cortex. Concerted reorganization of the actin cytoskeleton appears to regulate the membrane potential and the alignment of CG beneath the plasma membrane to facilitate the responses at egg activation: proper Ca^2+^ response to fertilizing sperm, CG exocytosis, as well as monospermic penetration, spindle formation, and polar bodies extrusions. Results on overripe starfish eggs that are fertilized in a time window different from their optimal have shown that the deterioration of the cells induces dramatic morphological alterations of the surface and cortex that negatively impact the fertilization process. The same changes appear to happen in mammalian oocyte ageing. In view of the difficulties of gamete availability and accessibility as well as the ethical issues involved, extensive studies on the reproductive biology of human oocytes has met with obstacles. Thus, animal models of starfish and sea urchin have invaluable practical merits.

In this review, we emphasize that one of the fundamental reactions of gametes at fertilization is concerted changes of the actin filaments in both gametes. For eggs, the cortical reaction is compromised when the egg is not healthy, thus a successful cortical reaction could be an indicator of the “quality” of the eggs, and the latter is mainly determined by the proper regulation of the actin cytoskeleton. Thus, technical aspects of ART to preserve functionality of the actin cytoskeleton in the human eggs would be also important. The usage of marine eggs as experimental systems to study oocyte maturation and fertilization is also advantageous in that perspective.

## Data Availability

Not applicable.
